# Identification of homozygosity-rich regions in the Holstein genome

**DOI:** 10.18699/VJGB-23-57

**Published:** 2023-09

**Authors:** M.G. Smaragdov

**Affiliations:** Russian Research Institute of Farm Animal Genetics and Breeding – Branch of the L.K. Ernst Federal Science Center for Animal Husbandry, St. Petersburg, Pushkin, Russia

**Keywords:** ROH, SNP, inbreeding, cattle, ROH, SNP, инбридинг, крупный рогатый скот

## Abstract

In this study, 371 Holstein cows from six herds and 26 Holstein bulls, which were used in these herds, were genotyped by the Illumina BovineSNP50 array. For runs of homozygosity (ROH) identification, consecutive and sliding runs were performed by the detectRUNS and Plink software. The missing calls did not significantly affect the ROH data. The mean number of ROH identified by consecutive runs was 95.4 ± 2.7, and that by sliding runs was 86.0 ± 2.6 in cows, while this number for Holstein bulls was lower 58.9 ± 1.9. The length of the ROH segments varied from 1 Mb to over 16 Mb, with the largest number of ROH having a length of 1–2 Mb. Of the 29 chromosomes, BTA 14, BTA 16, and BTA 7 were the most covered by ROH. The mean coefficient of inbreeding across the herds was 0.111 ± 0.003 and 0.104 ± 0.004 based on consecutive and sliding runs, respectively, and 0.078 ± 0.005 for bulls based on consecutive runs. These values do not exceed those for Holstein cattle in North America. The results of this study confirmed the more accurate identification of ROH by consecutive runs, and also that the number of allowed heterozygous SNPs may have a significant effect on ROH data.

## Abstract (ru)

В настоящем исследовании 371 корова голштинской породы из шести стад и 26 быков голштинской породы, которые использовались в этих стадах, были генотипированы с помощью чипа llumina BovineSNP50. Для идентификации гомозиготных последовательностей (ROH) выполнялись последовательные и скользящие сканирования с помощью программ detectRUNS и Plink. Пропущенные SNP генотипы не оказали существенного влияния на данные ROH. Среднее количество RОН, выявленное у коров при последовательных сканированиях, составило 95.4 ± 2.7, при скользящих сканированиях – 86.0 ± 2.6, тогда как у быков голштинской породы оно было меньше – 58.9 ± 1.9. Длина сегментов ROH варьировала от 1 до 16 Мб и более, при этом основное количество ROH имело длину 1–2 Мб. Из 29 хромосом наиболее насыщены ROH оказались ВТА 14, ВТА 16 и ВТА 7. Средний коэффициент инбридинга по стадам составил 0.111 ± 0.003 и 0.104 ± 0.004 на основе последовательных и скользящих сканирований соответственно, а для быков на основе последовательных сканирований – 0.078 ± 0.005. Эти значения не превышали показатели для голштинского скота в Северной Америке. Результаты исследования подтвердили более точную идентификацию ROH последовательными сканированиями, а также то, что количество разрешенных гетерозиготных SNP в ROH может оказывать существенное влияние на данные ROH

## Introduction

Inbreeding in dairy cattle is an inevitable phenomenon of artificial selection. Traditionally, the inbreeding coefficient is calculated based on ancestry (Meuwissen, Luo, 1992). With the advent of SNPs arrays (Matukumalli et al., 2009), it became possible to investigate autozygosity at a previously unattainable level (Peripolli et al., 2016). In fact, due to the runs of homozygosity (ROH) approach, animal genome analysis for long continuous homozygous stretches is still ongoing. The primary cause of autozygosity in livestock measured by ROH is assumed to be inbreeding (Peripolli et al., 2016) or consanguineous marriage in humans (Ceballos et al., 2018b). For identifying ROH, software based either on identity by descent (IBD) GERMLINE (Gusev et al., 2009), or on Hidden Markov Model (HMM) Beagle (Browning S., Browning B., 2010) and BCFtools (Narasimhan et al., 2016) has been elaborated. In addition, software based on scanning by SNPs window Plink (Purcell et al., 2007), overlapping sliding window SNP101 (Forutan et al., 2018), or both consecutive and sliding runs detectRUNS (Biscarini et al., 2018), as well the software based on other scripts (Howard et al., 2015; Kim et al., 2015), cgaTON (Zhang L. et al., 2013) have been provided. The commercial software SNP & Variation Suite (Golden Helix SNP & Variation Suite) is also widely used.

It has been shown that software based on HMM and IBD is inferior to other software mentioned above (Howrigan et al., 2011). The main challenge facing scientists is the lack of consistent criteria among studies regarding a threshold value of each parameter analyzed to determine ROH (Peripolli et al., 2016). The most crucial parameters that are used in any software are the number of heterozygous or missing SNP calls allowed in ROH. There is an inconsistency between thresholds that should be applied in studies. Some authors disallowed any number of heterozygous SNPs in ROH (Ferencakovic et al., 2011; Purfield et al., 2012; Bjelland et al., 2013; Marras et al., 2014), others allowed one, two and more heterozygous SNPs depending on the length of the ROH segments (Ferenčaković et al., 2013; Karimi, 2013; Zavarez et al., 2015; Zhang Q. et al., 2015a; Mastrangelo et al., 2016; Ceballos et al., 2018a; Addo et al., 2019; Zinovieva et al., 2020). Anyway, M. Ferenčaković et al. (2013) suggested that allowing a certain amount of genotype errors in a long ROH could minimize the underestimation of these segments. Although S. Mastrangelo et al. (2016) showed different values of the inbreeding coefficient, if heterozygous genotypes were allowed.

There are relatively few studies assessing which set of these parameters is optimal for detecting ROH, in order to better understand their effect on identified autozygosity. M. Ferenčaković et al. (2013) have shown that SNP array density and genotyping errors introduce patterns of bias in the assessment of autozygosity. These authors observed that allowing heterozygous SNPs in ROH can lead to the merging of adjacent ROH segments which resulted in biased estimates of the ROH number. Based on simulation data, D. Howrigan et al. (2011) recommended disallowing existence of any heterozygous SNPs in ROH. Summarizing, there is currently no consensus on a reasonable number of heterozygous SNPs in ROH to avoid bias in the ROH data.

When planning this study, special attention was paid to assessing the impact of allowed missing SNPs and heterozygous SNPs in ROH runs on the results using commonly applied consecutive and sliding runs. Another goal of the study was to evaluate the distribution of ROH in the chromosomes, and the effect of allowed heterozygous SNPs on inbreeding scores.

The following main objectives of the study were: (i) to assess the number and length of ROH segments in the cows and bulls genome, as well as their proportion in the chromosomes, (ii) to calculate the inbreeding coefficient, (iii) to assess the data bias resulting from an allowance of missing and heterozygous SNPs in ROH, (iv) to use the sliding windows and consecutive runs to obtain ROH data.

## Materials and methods

Animal resources and SNPs genotyping. Data and genotypes were obtained from Committee on Agro-Industrial Complex of the Leningrad region. This study analyzed Holstein cows born from 2010 to 2013 in six herds located in the Leningrad region (Russia). More information on breeding our local Holstein cattle can be found in the article (Kudinov et al., 2022).

Animals for genotyping were selected by farmers with regard to the pedigree structure of the herd. The sampled animals accounted for 8–15 % of the total number of dairy cows in herds. Altogether, 371 cows from six herds and 26 bulls from the Netherlands, North America, Germany and Canada used in these herds were genotyped by BovineSNP50 v. 2.0 array (Illumina, USA). Quality control was carried out by Plink. (i) SNPs calls with a quality score of less than 0.7 were removed. (ii) Only autosomal chromosomes were considered. (iii) 5 % of missed SNPs and 1 % MAF were allowed, which resulted in 48,108 SNPs for cows and 43,441 for bulls. Total genotyping rate was > 0.99.

Identification of ROH. The ROH segments were identified using detectRUNS (Biscarini et al., 2018) implemented in the R environment (http://www.r-project.org/index.html), and Plink tool (Purcell et al., 2007). The parameters applied to define ROH by detectRUNS for consecutive runs method were: (i) the minimum number of SNPs required to define segments as ROH, 15 and 20, (ii) the number of missing calls allowed in a ROH segment, 0–4, (iii) the number of heterozygous calls allowed in a ROH segment, 0–2, (iv) the minimum length of ROH segments, 250 Kb, (v) the maximum gap between ROH segments, 1 Mb.

For sliding window method in detectRUNS the parameters and thresholds were: (i) window size 15 and 20 SNPs, (ii) the threshold 0.05, (iii) the minimum number of SNPs required to define segments as ROH, 15 and 20, (iv) the number of missing calls allowed in a ROH segment, 0–4, (v) the number of heterozygous calls allowed in a ROH segment, 0–2, (vi) the minimum length of ROH, 250 Kb, (vii) the maximum gap between ROH segments, 1 Mb, (viii) the minimum allowed density of SNPs, 1 SNP per 1 Mb.

The parameters applied to define ROH by Plink were (i) the sliding window, 20 SNPs, (ii) the proportion of homozygous overlapping windows, 0.05, (iii) the minimum number of SNPs in ROH, 20, (iv) the density was one SNP per 60 Kb, (v) the number of missing SNPs was zero, (vi) the number of heterozygous SNPs was zero.

Inbreeding coefficients (FROH) were calculated as the sum of the animal’s ROH lengths divided by the total length of the autosomes covered by SNPs (2508.706681 Mb).

## Results

Impact of missing SNPs on ROH data. Primarily, the effect of missing SNPs allowed in ROH on the data was evaluated by consecutive and sliding runs. No impact on ROH data was found for either method if one to four missing SNP calls were allowed in ROH. Therefore, to further evaluate the ROH results, this value was set to zero.

Effect of heterozygous SNPs on ROH data based on consecutive runs. To evaluate the number of ROH segments in the cow genome, 15 SNPs (Suppl. Material 1)1 and 20 SNPs (Table 1) consecutive runs were carried out. When ROH segments were not interrupted by heterozygous SNPs, the mean number of ROH was 1.9 times greater at 15 SNPs runs ( p ≤ 0.03). In fact, the average number of ROH across the herds was 182.1 ± 3.4 at 15 SNPs runs compared to 95.4 ± 2.7 at 20 SNPs runs. To avoid overestimation of the autozygous ROH due to short ROH segments, 20 SNPs runs were used further.

Supplementary Materials are available in the online version of the paper: https://vavilovj-icg.ru/download/pict-2023-27/appx17.pdf


**Table 1.**
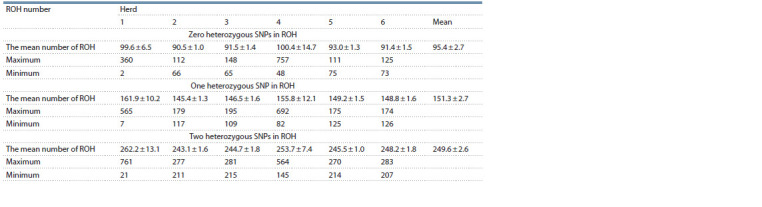
Estimated mean ROH number (± SE) in the herds based on 20 SNPs consecutive runs (detectRUNS)

For an adequate understanding of the results, it is necessary to define the term ROH further used. ROH is a contiguous homozygous SNP sequence uninterrupted by heterozygous SNPs, except for the allowed number of heterozygous SNP. Descriptive data statistics are given in Table 1. The mean number of ROH varied across herds. However, the differences between them are insignificant (t-test). It should be noted that there is considerable variation in the ROH number among the fourth herd cows. This result was due to a large number of ROH in one cow (757 ROH segment). The exclusion of this cow resulted in the mean ROH of 85.9 ± 2.1 in the fourth herd. However, this did not lead to significant differences between the herds (t-test). The effect of allowed heterozygous SNPs on the number and length of the ROH segments was assessed when their values ranged from 0 to 2. Initially, the average number of ROH increased more than 1.6-fold from 95.4 ± 2.7 to 151.3 ± 2.7 when one heterozygous SNP in ROH was allowed (see Table 1). Then the mean increased to 249.6 ± 2.6 with an increase in the number of allowed heterozygous SNPs in ROH to two. Thus, the allowance of heterozygous SNPs leads to a significant ( p ≤ 0.02) increase in the number of ROH.

The length of the ROH segments has been classified into five categories (1–2 Mb, 2–4, 4–8, 8–16, and >16 Mb). The most abundant in the number of ROH was the 1–2 Mb class (Suppl. Material 2). The largest proportion of the ROH number had the same class, up to two allowed heterozygous SNPs. A particularly noticeable increase in the number of ROH in this class occurred with the use of 15 SNP runs (see Suppl. Material 2). These data indicate the presence in the genome of cows of a large number of short (less than 1 Mb) ROH segments, which are more effectively detected when scanning for 15 SNPs.

ROH identification based on sliding runs. As for 15 SNPs and 20 SNPs, the sliding runs were used (Table 2). Interestingly, the data for 15 SNPs runs identified by both consecutive and sliding runs were largely the same (see Suppl. Material 1 vs. Table 2 (15 SNPs)), while for 20 SNPs consecutive and 20 SNPs sliding runs, the data differ somewhat, but insignificantly (t-test) (see Tables 1 and 2). To obtain a comparable result with consecutive runs, 20 SNPs window was used further. Descriptive statistic for 20 SNPs sliding data is given in Table 2. The mean number of ROH between herds was insignificant (t-test). But, similar to consecutive runs after exclusion of the most deviated cow (it included 731 ROH segments) among the fourth herd, the mean number of ROH became 77.6 ± 2.0. However, this value still did not significantly differ from those for other herds (t-test). The average number of ROH increased by 1.7 times, from 86.0 ± 2.6 to 146.7 ± 1.7, when one heterozygous SNP was allowed in ROH, then by 3 times when two heterozygous SNPs were allowed. The observed increase in the number of ROH was significant p ≤ 0.02 (t-test).

The length of the ROH segments for sliding runs has been classified into the same five categories (1–2, 2–4, 4–8, 8–16, and >16 Mb) as it has been carried out for consecutive runs (Suppl. Material 3). The most numerous in the number of ROH has occurred in the same class of 1–2 Mb, in which a considerable increase in ROH segments was observed with an increase in the number of allowed heterozygous SNPs in ROH. This indicates the proximity of numerous ROH segments shorter than 1 Mb in the cow genome.

ROH identification based on Plink. Plink software is widely used in ROH studies. Therefore, it is necessary to compare the data obtained by Plink and detectRUNS. The mean number of ROH obtained with Plink was 74.9 ± 1.9 and this value was no different from the value calculated by detectRUNS based on sliding runs 86.0 ± 2.6 (t-test). The fact that Plink identified fewer ROH segments mainly in the 1–2 Mb class than detectRUNS detected (see Suppl. Materials 2 and 3) indicates Plink’s lesser ability to identify short segments less than 1 Mb. Thus, the data obtained for the shortest ROH length class can be highly dependent on the software and parameters used.

**Table 2.**
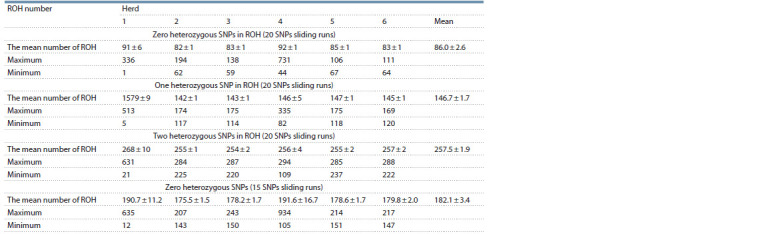
Estimated mean ROH number (± SE) across the herds based on sliding runs (detectRUNS)

Comparative analysis of consecutive and sliding runs. Comparative analysis of the consecutive and sliding data led to the following conclusions. When heterozygous SNPs were disallowed, the consecutive runs showed a slightly bigger mean number of ROH than sliding runs (94.4 ± 2.7 vs. 86.0 ± 2.6) and even bigger for sliding windows (Plink) 74.9 ± 1.9, but the difference between them was insignificant (t-test). The fewer SNPs were used in consecutive runs, the more 1–2 Mb ROH segments there were (Table 3). Summarizing the comparative analysis of the applied methods, one can come to the conclusion that there are some differences in the results obtained by these methods.

**Table 3.**
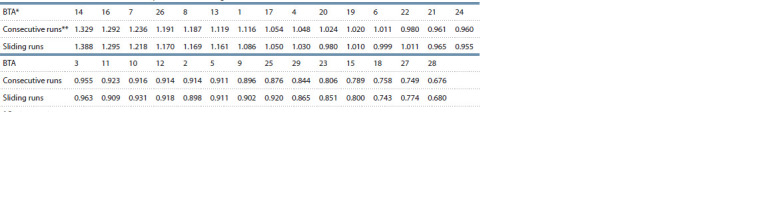
Rank of the cows chromosomes by their ROH coverage * Bos taurus autosome. ** The rank values were ranged from maximum to minimum only for consecutive runs.

Distribution of ROH in the cow chromosomes. To evaluate the chromosomes with the largest number of ROH segments taking into account their length, the following calculation was carried out. For each chromosome, the proportion of ROH in it was divided by the share of its size in the cattle genome. The rank chromosome calculation is shown in Suppl. Material 4. For both runs, the list of chromosomes ranked in this way is shown in Table 3. Out of 29 chromosomes, the top chromosomes covered with ROH were BTA 14, BTA 16 and BTA 7, not BTA 1 (seventh position in the list), BTA 2 (20th position in the list) and BTA 3 (16th position in the list). Thus, the number of ROH in the chromosomes was not proportional to their length. Spearman’s correlation between consecutive and sliding runs data in Table 3 was r = 1.0 ( p ≤ 2.0E–07). Whether this fact is a result of drift or/and selection requires further study.

Inbreeding. To assess the level of inbreeding in the herds, the mean inbreeding coefficient was calculated across all herds (Tables 4 and 5). When heterozygous SNPs were disallowed, the mean inbreeding coefficients across the herds amounted to 0.111 ± 0.003 and 0.104 ± 0.004 for consecutive and sliding runs, respectively, and the difference between them was insignificant (t-test). The mean inbreeding coefficient estimated by Plink was 0.105 ± 0.004, which is consistent with those for sliding runs. A greater variability in inbreeding occurred for the fourth herd. This result is mainly associated with a highly inbred cow in this herd. Exclusion of this cow results in the average inbreeding coefficient of 0.096 ± 0.005 and 0.089 ± 0.005 for consecutive and sliding runs. It should be noted that in this herd the cows were inseminated only from the Netherlands bulls, while in other herds the bulls’ semen from North America, Germany, Canada, and the Netherlands was used. The proportion of the bulls from these countries used in the herds was published in the article (Smaragdov et al., 2018). After excluding the highly inbred cow, the average inbreeding coefficient in the fourth herd decreased compared to other herds. This result indicates the correct selection of the bulls even if their semen was imported from the same country. The fourth herd deviated significantly from the other herds when variability was measured by the Wright’s fixation index or PCA (Smaragdov, Kudinov, 2020). When one heterozygous SNP was allowed in ROH, then the mean inbreeding coefficient across all herds was 0.145 ± 0.003 and 0.148 ± 0.003 based on consecutive and sliding runs. Thus, the allowance of even one heterozygous SNP resulted in an increase in the inbreeding coefficient ( p ≤ 0.06). Therefore, to assess inbreeding in the herds, heterozygous SNPs should be disallowed in ROH due to sizable bias.

**Table 4.**
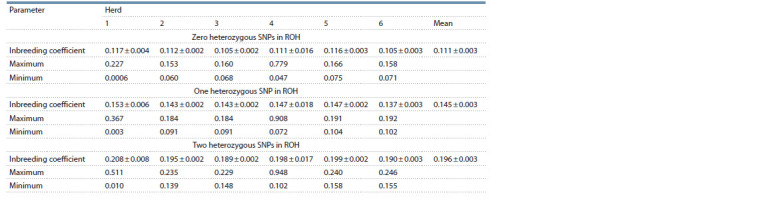
Estimated average inbreeding coefficient (± SE) in the herds based on 20 SNPs consecutive runs (detectRUNS)

**Table 5.**
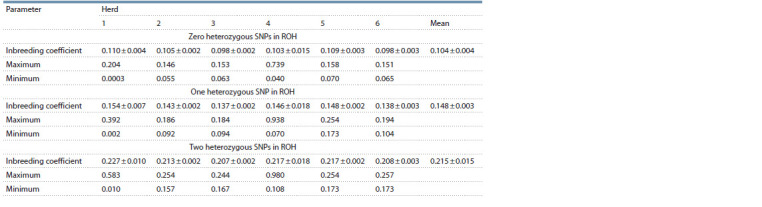
Estimated average inbreeding coefficient (± SE) in herds based on 20 SNPs sliding runs (detectRUNS)

Confirmation of results obtained on cows with data on bulls. To validate the results obtained on the cows, the bulls that have been used two generations ago in the same herds were analyzed for ROH. The mean number of the ROH segments for the bulls, 58.9 ± 1.9, turned out to be significantly less than for the cows, 95.4 ± 2.7 ( p ≤ 0.05) (Tables 1 and 6). The mean inbreeding coefficient for the bulls was 0.078 ± 0.005 and did not differ significantly from the cows (t-test). The coefficient of inbreeding did not significantly increase when one heterozygous SNP was allowed (t-test) (Table 7).

**Table 6.**
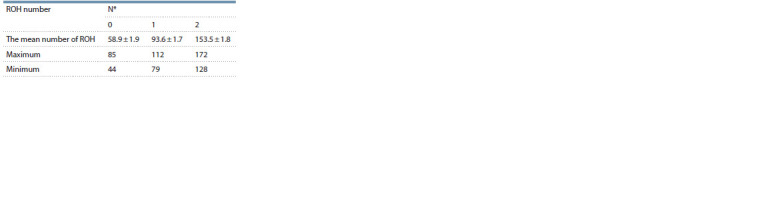
Estimated mean ROH number (± SE) on bulls based on 20 SNPs consecutive runs (detectRUNS) * The number of allowed heterozygous SNPs in ROH.

**Table 7.**
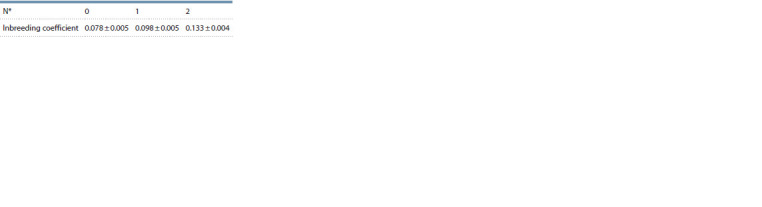
Estimated inbreeding coefficient (± SE) on bulls based on 20 SNPs consecutive runs (detectRUNS) * The number of allowed heterozygous SNPs in ROH.

## Discussion

Over the past decade, the runs of homozygosity approach has been widely used both in humans (Ceballos et al., 2018b) and farm animals (Peripolli et al., 2016). A distinctive feature of ROH studies is the variety of software and threshold criteria used in them. The most widely applied software tools for identifying ROH segments are either sliding window or consecutive runs. We preferred detectRUNS, where both approaches have been implemented (Biscarini et al., 2018).

The consecutive runs resulted in the average number of ROH 94.4 ± 2.7, while sliding runs, 86.0 ± 2.6. These values for North American (Forutan et al., 2018), Italian (Marras et al., 2014), European Holstein (Zinovieva et al., 2020), and Polish Holstein Black-and-White variety (Szmatoła et al., 2019) are 82.3 ± 9.8 (SD), 81.7 ± 9.7 (SD), 74.6 ± 2.3 (SE), and 53.3 ± 7.3 (SD) respectively. The first three values do not differ significantly from ours, while the value for Polish cattle differs considerably. It should be noted that the allowance of even one heterozygous SNP in ROH significantly increases the number of ROH by 55.9 and 60.7 points for consecutive and sliding runs, respectively (see Tables 1 and 2). A limited number of studies have analyzed the effect of allowed heterozygous SNPs on ROH data. D. Howrigan et al. (2011) recommended disallowing the use of any heterozygous SNPs in ROH, while M. Ferenčaković et al. (2013) suggested that the number of allowed heterozygous SNPs should be determined separately for each ROH length of interest and for each SNPs density. Moreover, the allowance of heterozygous SNPs in ROH leads to a sizable bias in the inbreeding coefficient (Mastrangelo et al., 2016). My results confirm this conclusion.

The relative frequency of the ROH number in different length classes obtained from the cows data for consecutive runs were 61.4 % (1–2 Mb), 19.8 % (2–4 Mb), 11.3 % (4–8 Mb), 5.5 % (8–16 Mb) and 1.9 % (longer than 16 Mb), while for sliding runs these values were 60, 19.8, 12.1, 5.8, and 2.1 %. Thus, the largest number of ROH was identified in the shortest 1–2 Mb class. Plink-running of the cows genome revealed the following ROH frequencies in five categories 52 % (1–2 Mb), 25 % (2–4 Mb), 14 % (4–8 Mb), 7 % (8–16 Mb) and 2.5 % (longer than 16 Mb), the distribution of which is slightly different from those defined by detectRUNS. For North American Holstein animals, these values were 43.5, 23.9, 17.7, 10.5, and 4.7 % (Forutan et al., 2018). The corresponding values for Italian Holstein bulls were 56.9, 20.8, 11.9, 7.2, and 3.7 % (Marras et al., 2014) and Polish Holstein, 23, 19, 9.8, 4.4, and 1.3 % (Szmatoła et al., 2019). Thus, when we used detectRUNS to scan the genome of our local Holstein cows, we obtained an abundant number of short ROH as a result of haplotypes reflecting the ancient relationship within breeding animals. But, when we used Plink, the values were similar to the American and Italian data. It should be noted that the authors of the article (Szmatoła et al., 2019) used the cgaTOH software and their data differ considerably from other data. Whether this result was due to the cgaTOH software (minimal number of 30 consecutive homozygous SNPs in ROH) or/and selection requires further analysis. Estimation of the true number of short ROH is important, since 0.1–3 Mb ROH segments have the more number of deleterious variants than segments longer than 3 Mb (Zhang Q. et al., 2015b). For evaluation of the genomic estimated breeding value (GEBV), short ROH is essential for genomic construction of ROHbased relationship matrix (GROH) (Luan et al., 2014).

According to my data, the largest number of ROH falls into the 1–2 Mb class. As the number of allowed heterozygous SNPs in ROH increases, the number of ROH segments in the shortest 1–2 Mb class increases as well (see Suppl. Materials 2 and 3). This fact indicates a close location of a large number of short, less than 1 Mb, ROH segments

The same conclusion was reached in a study of ten sequenced (WGS) breeds of cattle (Mulim et al., 2022). Then, the results of the animals ROH genome scanning can substantially depend not only on the selection but also on the genotyping method and the software used to identify short ROH segments. This fact should be taken into account in the comparative analysis of the ROH data

Estimated by detectRUNS, the mean inbreeding coefficient for six herds was 0.111 ± 0.003 and 0.104 ± 0.004 for consecutive and sliding runs, respectively, and for bulls, 0.078 ± 0.005 for consecutive runs. It was equal to 0.105 ± 0.004 based on the sliding window runs evaluated by Plink. It should be noted that cows from six herds did not differ in the mean inbreeding coefficient (see Tables 4 and 5), while according to Principal Components Analysis, the fourth herd differed significantly from all other herds (Smaragdov, Kudinov, 2020). Therefore, this difference is not due to inbreeding

The accurate knowledge of inbreeding in the herds that occurred several decades in the past is necessary both for calculating the inbreeding trend and for evaluating selection strategies. To solve this problem, high-density arrays or whole genome sequencing (WGS) should be used. Comparison of 50k and HD panels provides evidence that the data from the 50k panel lead to imprecise determination of short ROH segments (Ferenčaković et al., 2013). However, it has been shown that ROH detection based on high-density or 50k array data might give the estimates of current inbreeding most similar to ROH values obtained from the sequence data (Zhang Q. et al., 2015a). M. Bhati et al. (2020) provided comprehensive WGS data for Braunvich cattle. Medium-sized ROH (0.1–2 Mb) were the most frequent class (50.46 %) and made the largest contribution (75 %) to total genomic inbreeding, while short, 50–100 Kb, ROH occurred almost as frequently (49.17 %) as medium-sized ROH, they contributed only 19.52 % to total genomic inbreeding. These findings provide an accurate estimate of short ROH in the cattle genome and their contribution to total inbreeding. The average FROH estimated from the WGS data was 0.14 in Braunvich cattle. This value is less than WGS FROH in Holstein, 0.18 (Bhati et al., 2020). Summarizing, the 50k panel cannot accurately capture ancient inbreeding that occurred a few decades in the past. The inbreeding coefficient of American Holstein measured with ROH in 2011 was 0.12 and after applying genomic selection, it increased to 0.15 in 2018 (Forutan et al., 2018). For European (Zinovieva et al., 2020), Italian (Marras et al., 2014), and Polish Holstein (Szmatoła et al., 2019), these values were 0.108 ± 0.006 (SE), 0.116 ± 0.001 (SE), and 0.118 ± 0.027 (SD), respectively. It is important to note that in the above studies, ROH data were based on the 50k array; thereby, ROH segments not shorter than 1 Mb were identified. Once again, we have to admit that, according to our data, an increase in the number of mostly short ROHs (1–2 Mb) by 395 points identified during consecutive runs compared to sliding runs (Suppl. Materials 2 and 3) leads to only a slight increase in the inbreeding coefficient (Tables 4 and 5).

It can be assumed that there should be an event horizon for a herd or population, beyond which it is impossible to obtain valid information about inbreeding events in history of their breeding. I hypothesize that in our local population, a reduced effective population size, ongoing admixture and inbreeding throughout its history, accompanied by recombination, should lead to the largest number of short ROH less than 1 Mb in the herds currently studied. These short ROH can be considered as ancient ROH segments formed by some population events, such as drift, bottleneck, and inbreeding that occurred many decades ago. The bottleneck in our local herds has not previously been proven by Principal Component Analysis (Smaragdov, Kudinov, 2020). An accurate interpretation of these short ROH can be troublesome without knowledge of the herd management history. In addition, it is very important to know the true number of short ROHs in the analyzed animals resulting from inbreeding (see above-mentioned WGS data). Thus, the event horizon can depend on both pedigree information, ROH length profile (SNPs array or WGS used) as well as on the algorithm-defined approach to ROH identification. However, ROH segments shorter than 500 Kb can be considered to be beyond the event horizon due to strong LD and inconsistency with autozygosity. The short ROH characterized by strong LD among markers are not always considered autozygous, but nevertheless they may have formed due to mating with distantly related animals (McKay et al., 2007). Summarizing, it should be assumed that inbreeding data can be only relatively correct based on ROH larger than 1 Mb (no more than 50 generation back)

A number of studies have noted an uneven distribution of ROH in the bovine genome, e. g. (Ferencakovic et al., 2011; Sölkner et al., 2014; Howard et al., 2015). Giving the number of ROH in the chromosomes, we calculated their rank taking into account the proportion of chromosome length in the genome of the cattle (see Table 3). Out of 29 chromosomes, the most covered with ROH segments were BTA 14, BTA 16 and BTA 7 for both approaches used. D. Purfield et al. (2012) noticed that among the breeds studied, BTA 14 and BTA 16 had the highest degree of ROH segments overlap. The regions of the genome with the highest frequency of occurrence of ROH in the genome of the studied animals were called “ROH islands” (Nothnagel et al., 2010; Pemberton et al., 2012). The ROH islands on BTA 14 and BTA 16 were identified among Polish Holstein-Friesian animals (Szmatoła et al., 2019). In Holstein cows in our study, ROH islands were localized in BTA 7 and BTA 14 (unpublished results). In American Holstein, ROH distribution was more variable among the genomes of the selected animals, compared to a relatively even ROH distribution in unselected animals (Kim et al., 2013). Regions with a high proportion of ROH for American and New Zealand Jersey cows and bulls were revealed on BTA 3 and BTA 7 (Howard et al., 2015). On BTA 14 and BTA 16, one strongest ROH region was found common for Kholmogor and Holstein breeds and one region common for Yaroslavl and Holstein breeds (Zinovieva et al., 2020). Extremely non-uniform ROH patterns among bovine populations of Angus, Brown Swiss, and Fleckvieh breeds were mainly located on BTA 6, BTA 7, BTA 16, and BTA 21 (Sölkner et al., 2014). The highest number of ROH islands among all Neilore breed lineages was found on BTA 7 (Peripolli et al., 2018a). In addition, an enrichment of genes affecting traits of interest for dairy breeds was shown on BTA 14 in dairy Gyr breed (Bos indicus) (Peripolli et al., 2018b). D. Goszczynski et al. (2018) analyzed ROH >16 Mb (three generations from a common ancestor) in highly inbred Retinta bulls. Among other chromosomes, the highest occurrence of ROH was found on BTA 7. Summarizing the above studies, it can be suggested that BTA 7 is outstanding regarding ROH islands occurrence in the cattle genome but in general there is no overall direct relationship between the proportion of ROH segments in the chromosomes and ROH islands identified there.

As discussed above, the number of identified short ROH is highly dependent on the software used and also on the genotyping method. Moreover, it can be suggested that consecutive runs more accurately identified the ROH pattern in the cow genome. However, both methods coincide in assessing the distribution of ROH segments on chromosomes (see Table 3). Taking the findings together, it should be assumed that uneven distribution of ROH segments in the cow genome is a result of different inbreeding events that have occurred in their history.

## Conclusion

Analysis of ROH data showed that consecutive runs most accurately identified ROH in the cattle genome. It has been shown that missing SNPs did not have a noticeable effect on the number of ROH, while an allowance of even one heterozygous SNP in the ROH segments had a significant effect. Therefore, care should be taken to allow any heterozygous SNPs in the ROH. The average number of ROH across herds was 95.4 ± 2.7 and their length varied from 1 Mb to more than 16 Mb. The class with the length of 1–2 Mb was the most numerous in the number of ROH. This confirms the long history of inbreeding in herds for many decades in the past. Moreover, the number of ROH in the chromosomes does not depend on their length. ROH segments mainly cover BTA 14, BTA 16, and BTA 7. The average inbreeding coefficient for our local Holstein herds was 0.111 ± 0.003, which is not much different from the Holstein cattle inbreeding coefficient worldwide. This value indicates competent management of the studied herds. In addition, the inbreeding coefficient obtained on cows is consistent with the inbreeding coefficient of 0.078 ± 0.005 calculated in our study for Holstein bulls from other countries. These bulls have been used in breeding our local Holstein cattle two generations ago.

## Conflict of interest

The authors declare no conflict of interest.
